# Beyond motor recovery: Stem cell‐derived exosomes for sensory restoration and neuropathic pain relief after spinal cord injury

**DOI:** 10.1002/ibra.70031

**Published:** 2026-09-26

**Authors:** Md Miftahul Mithu, Sahiduzzaman, Shirsendu Narayan Roy, John Jacob Mathew, Devunipalli Karthik Haraveer, Basundhari Thongbam, Kancharllapalli Prasanna Kumar, Sourov Dey, Md Arifuzzaman

**Affiliations:** ^1^ Department of Ilizarov Surgery and Deformity Correction Bangladesh Center for Ilizarov Surgery Kushtia Bangladesh; ^2^ Department of Microbiology and Infectious Diseases Prafulla Chandra Sen Medical College & Hospital Arambag West Bengal India; ^3^ Department of Emergency Medicine Harirampur Rural Hospital Dakshin Dinajpur West Bengal India; ^4^ Department of Ophthalmology Christian Medical College & Hospital Ludhiana Punjab India; ^5^ Department of Pediatrics and Neonatology Prasad Hospitals Hyderabad India; ^6^ Department of Community Medicine Jawaharlal Nehru Hospital Imphal Manipur India; ^7^ Department of Psychiatry Konaseema Institute of Medical Science and Research Foundation (KIMS) Amalapuram India; ^8^ Department of Medicine The Holy Family Red Crescent Medical College Hospital Dhaka Bangladesh; ^9^ Department of Ilizarov and Deformity Correction Magura Medical College Hospital Magura Bangladesh

**Keywords:** motor function, neuropathic pain, sensory function, spinal cord injury, stem cell‐derived exosomes

## Abstract

Spinal cord injury (SCI) causes persistent neurological deficits that extend beyond motor impairment, with sensory dysfunction and neuropathic pain substantially compromising quality of life. Stem cell‐derived exosomes have emerged as promising cell‐free therapeutic agents because they deliver diverse bioactive molecules, including proteins, microRNAs, and long non‐coding RNAs, while retaining many of the paracrine benefits of stem cells. This review summarizes recent advances in the application of stem cell‐derived exosomes for sensory restoration and neuropathic pain relief after SCI, with particular emphasis on their underlying mechanisms and emerging delivery strategies. We first discuss exosome biogenesis, molecular composition, intercellular communication, and engineering approaches. We then summarize their established roles in motor recovery through neuroprotection, immunomodulation, axonal regeneration and myelin repair. Importantly, we highlight emerging evidence that stem cell‐derived exosomes may restore sensory function by promoting axonal regeneration, synaptic remodeling, neural differentiation, and glial metabolic reprogramming. In parallel, exosomes alleviate neuropathic pain by suppressing microglial and astrocytic activation, modulating TLR/MyD88/NF‐κB signaling, reducing neuroinflammation and pathological nociceptive signaling, and promoting remyelination and tissue repair. Exosomes also preserve blood‐spinal cord barrier integrity through regulation of endothelial cells, pericytes, matrix metalloproteinases, and vascular signaling, for both motor and sensory recovery. Finally, we discuss challenges related to exosome heterogeneity, manufacturing standardization, delivery, safety, and limited sensory‐specific evidence, and highlight engineering and biomaterial‐based strategies for future clinical translation. Overall, stem cell‐derived exosomes represent a multifunctional therapeutic platform with potential to move SCI treatment beyond motor recovery toward comprehensive sensory restoration and pain management.

## Graphical abstract

Stem cell‐derived exosomes offer a promising cell‐free therapy for spinal cord injury by delivering diverse bioactive cargos. This review highlights their roles in motor recovery, sensory restoration, and neuropathic pain relief through axonal regeneration, synaptic remodeling, neural differentiation, glial metabolic reprogramming, immunomodulation, remyelination, and blood‐spinal cord barrier protection. Emerging engineering and biomaterial‐based delivery strategies may enhance therapeutic efficacy while addressing exosome heterogeneity, manufacturing, safety, delivery, and limited sensory‐specific evidence remains essential for clinical translation.

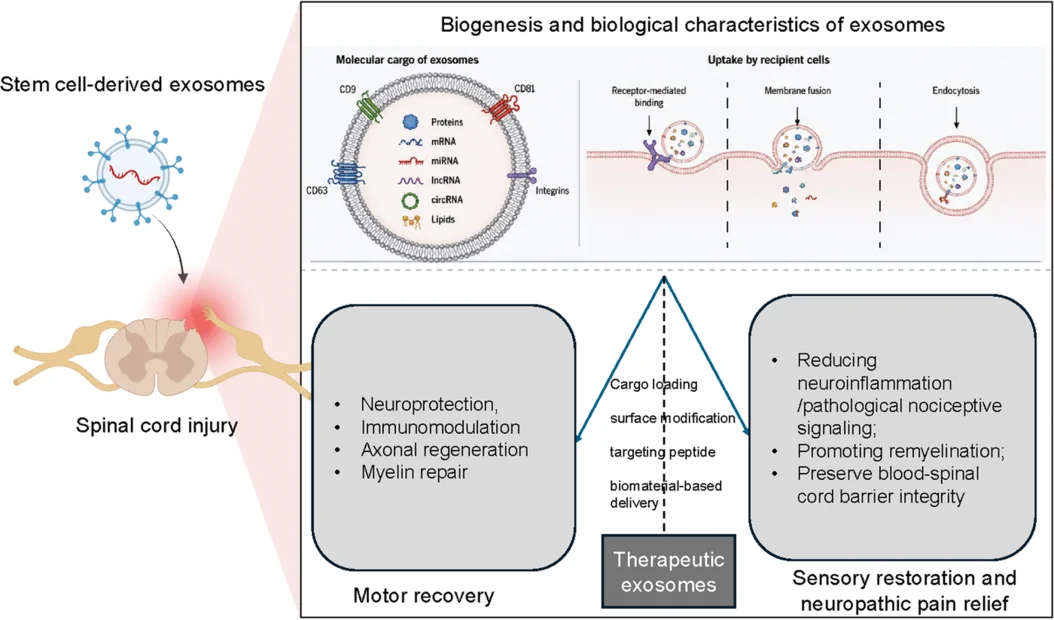

## 1 INTRODUCTION

Spinal cord injury (SCI) is a traumatic event that often leads to profound neurological deficits and functional impairment.^1^ The pathological process following SCI is classically divided into primary mechanical injury and secondary cascade reactions. Secondary damage—including local ischemia, oxidative stress, inflammatory cell infiltration, and glial scar formation—collectively establishes a hostile microenvironment that impedes neural repair and is the primary cause of poor nerve regeneration.^2^ Despite advances in clinical management, effective strategies for promoting neural regeneration and restoring neurological function remain limited. For example, neuroprotective agents such as riluzole may help improve neurological recovery, further large‐scale evidence is needed.^3^

Stem cell therapies, particularly those using mesenchymal stem cells (MSCs), have demonstrated therapeutic efficacy in animal models. However, challenges such as tumorigenicity, low cell survival rates, and ethical concerns remain unresolved.^4^ In this context, exosomes—nanoscale extracellular vesicles secreted by stem cells—have attracted considerable attention. Exosomes carry bioactive cargo including membrane proteins, cytosolic proteins, lipids, messenger RNAs (mRNAs), microRNAs (miRNAs), long non‐coding RNAs (lncRNAs), and other regulatory molecules,^5^ enabling them to recapitulate many of the paracrine therapeutic effects of their parent cells with greater safety. As for SCI, stem cell‐derived exosomes have been extensively investigated as a cell‐free therapeutic strategy, with numerous studies demonstrating improvements in motor function in animal models.^6^, ^7^ However, most of this research has focused on motor recovery, while the potential of exosomes to address sensory dysfunction and chronic pain has received comparatively less attention. Sensory loss and neuropathic pain are common and debilitating consequences of SCI that substantially impair quality of life, yet their restoration and relief remain major therapeutic challenges.^8^ Although studies specifically examining the effects of stem cell‐derived exosomes on sensory restoration and neuropathic pain relief after SCI are relatively limited, emerging evidence suggests that these vesicles may exert beneficial effects on sensory pathways and pain‐related mechanisms.^9^, ^10^ Accordingly, their potential to improve sensory function and alleviate neuropathic pain warrants greater attention, as these outcomes are essential for a more comprehensive assessment of functional recovery after SCI.

This review aims to summarize the emerging therapeutic potential of stem cell‐derived exosomes for sensory restoration and neuropathic pain relief after SCI. We first outline their biogenesis, biological characteristics, and therapeutic mechanisms, followed by an overview of their established effects on motor recovery. Particular emphasis is placed on their roles in restoring sensory function and alleviating neuropathic pain. Finally, current challenges in exosome standardization, delivery, safety, and clinical translation are discussed, together with emerging strategies involving exosome engineering and biomaterial‐based delivery. This review highlights the potential of stem cell‐derived exosomes to expand SCI treatment beyond motor recovery toward comprehensive neurological rehabilitation.

## 2 BIOGENESIS AND BIOLOGICAL CHARACTERISTICS OF EXOSOMES

Exosomes are nanoscale extracellular vesicles that mediate intercellular communication by transferring biologically active molecules between donor and recipient cells. Typically ranging from approximately 40 to 160 nm in diameter, exosomes are generated through the endosomal system and are released into the extracellular space after fusion of multivesicular bodies with the plasma membrane. Unlike free soluble factors, exosomes provide a membrane‐enclosed environment that protects their molecular cargo from extracellular degradation and facilitates its delivery to recipient cells.^11^ These properties have attracted increasing interest in exosomes as endogenous mediators of tissue repair and as cell‐free therapeutic platforms for neurological diseases, including SCI.

### 2.1 Biogenesis and molecular composition of exosomes

Exosome biogenesis begins with the inward budding of the plasma membrane to form early endosomes, which subsequently mature into late endosomes. Further inward budding of the endosomal membrane generates intraluminal vesicles within multivesicular bodies (MVBs). MVBs can either undergo lysosomal degradation or fuse with the plasma membrane, releasing their intraluminal vesicles into the extracellular space as exosomes. This process is regulated by several molecular machineries, including the endosomal sorting complex required for transport (ESCRT), as well as ESCRT‐independent mechanisms involving tetraspanins, lipids, and other membrane‐associated proteins.^5^

The molecular composition of exosomes reflects both the characteristics of the donor cell and the cellular state under which the vesicles are produced. Exosomes contain membrane proteins, cytosolic proteins, lipids, mRNAs, miRNAs, lncRNAs, and other regulatory molecules^5^ (Figure 1A). Their lipid‐enriched membrane contributes to structural stability, whereas surface proteins can influence cellular uptake and tissue targeting.^12^ Importantly, exosomal cargo is not simply a random representation of donor‐cell contents but can be selectively enriched through regulated cargo‐sorting mechanisms. Consequently, changes in the physiological or pathological state of donor cells may substantially alter the biological activity of the resulting exosomes.

**Figure 1:**
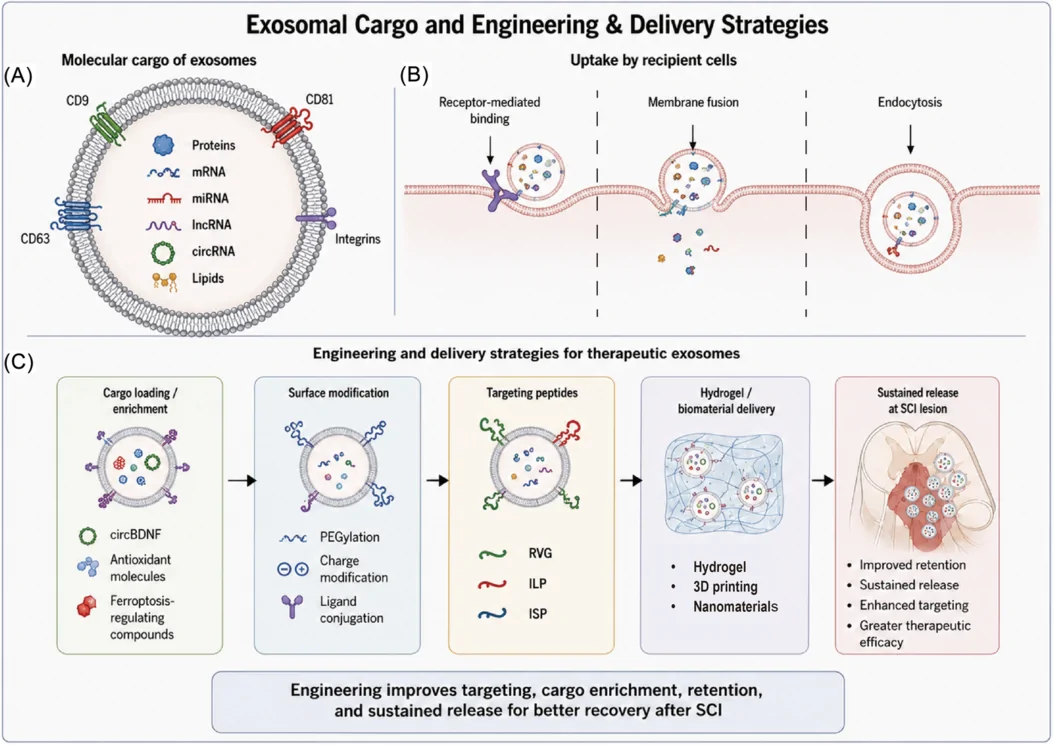
Exosomal cargo, intercellular communication and engineering and delivery strategies for therapeutic exosomes. (A). Exosomes contain membrane proteins, cytosolic proteins, lipids, mRNAs, miRNAs, lncRNAs, and other regulatory molecules. (B). Following release from donor cells, exosomes can interact with recipient cells through receptor‐mediated binding, membrane fusion, or endocytic pathways, allowing their cargo to influence gene expression and intracellular signaling. (C). Therapeutic exosomes can be optimized through cargo loading or enrichment, surface modification, targeting peptide conjugation, and biomaterial‐based delivery. These strategies enhance tissue targeting, therapeutic cargo enrichment, local retention, and sustained release, thereby improving exosome bioavailability and therapeutic efficacy for SCI repair. circBDNF, circular brain‐derived neurotrophic factor; ILP, insulin‐like peptides; ISP, intracellular sigma peptide; RVG, rabies viral glycoprotein; SCI, spinal cord injury.

This dynamic nature of exosome composition is particularly relevant to SCI, where the therapeutic effects of stem cell‐derived exosomes depend on the molecular repertoire inherited from their parent cells. MSC‐derived exosomes, for example, contain multiple proteins and regulatory RNAs capable of modulating inflammation, oxidative stress, neuronal survival, angiogenesis, and tissue remodeling.^13^ Thus, the therapeutic potential of exosomes is closely linked to both their intrinsic biological properties and the specific cargo delivered to recipient cells.

### 2.2 Exosomal cargo and intercellular communication

The ability of exosomes to transport multiple classes of bioactive molecules represents one of their most important biological characteristics. Following release from donor cells, exosomes can interact with recipient cells through receptor‐mediated binding, membrane fusion, or endocytic pathways, allowing their cargo to influence gene expression and intracellular signaling^14^ (Figure 1B). Through this mechanism, exosomes can establish functional communication between neurons, glial cells, endothelial cells, immune cells, and other components of the injured spinal cord.

Among the various cargoes, miRNAs have attracted particular attention because of their ability to regulate multiple target genes simultaneously. miRNAs are short non‐coding RNAs that generally suppress gene expression by binding to complementary sequences in target mRNAs.^15^ Exosomal transport provides an efficient mechanism for delivering miRNAs to recipient cells and thereby modifying cellular phenotypes at a distance. This feature is particularly relevant to SCI, in which multiple pathological processes, rather than a single molecular abnormality, contribute to secondary injury and impaired neural repair.

A comprehensive miRNA profiling study revealed extensive dysregulation of miRNAs associated with SCI, with the miR‐17‐92 cluster emerging as an important regulatory network involved in neurogenesis, synaptic activity, and cell survival. Integrative miRNA–mRNA–protein analysis further identified several potential therapeutic miRNAs, including miR‐20a. In vitro inhibition of miR‐20a promoted neurogenesis and reduced apoptosis under oxidative stress.^16^ Although this study did not necessarily establish all of these miRNAs as exosome‐specific cargoes, it illustrates the broader regulatory potential of miRNAs within the molecular environment of SCI and provides a rationale for exploiting exosome‐mediated RNA delivery to modulate these pathways.

The therapeutic relevance of exosomal RNA cargo can be further demonstrated by engineering exosomes to enrich specific neuroprotective molecules. For example, circular brain‐derived neurotrophic factor (circBDNF)‐loaded exosomes (circBDNF‐Exos) were generated using transfected HEK293T cells and evaluated in both an oxygen‐glucose deprivation/reoxygenation‐induced neuronal injury model and a T10 SCI mouse model. In vitro, circBDNF‐Exos increased BDNF expression and neuronal viability while reducing reactive oxygen species (ROS) accumulation, inflammation, and apoptosis. In vivo, treatment improved motor function, gait coordination, and motor‐evoked potentials and was accompanied by reduced oxidative stress and apoptosis and enhanced axonal regeneration.^17^ Mechanistically, circBDNF‐Exos activated the BDNF receptor TrkB and its downstream PI3K/AKT/mTOR signaling pathway, suggesting that exosome‐mediated delivery of neurotrophic cargo can simultaneously regulate neuronal survival and regenerative signaling.^17^

These findings highlight an important advantage of exosomes over single‐molecule therapies. Because exosomes naturally contain multiple classes of bioactive molecules and can be engineered to enrich specific therapeutic cargoes, they have the potential to simultaneously modulate several pathological processes. This multimodal capacity is particularly valuable after SCI, where inflammation, oxidative stress, neuronal apoptosis, axonal degeneration, demyelination, and vascular dysfunction occur concurrently.

### 2.3 Engineering and delivery strategies for therapeutic exosomes

Despite their favorable biological properties, the therapeutic application of exosomes faces several challenges, including rapid clearance, limited accumulation at the injury site, heterogeneous biodistribution, and insufficient retention within the damaged spinal cord. Engineering exosomes and combining them with biomaterial‐based delivery systems have therefore emerged as important strategies for improving therapeutic efficiency.

Hydrogels are particularly attractive for exosome delivery because they can provide a three‐dimensional matrix that retains exosomes within the lesion environment and enables sustained or controlled release. This approach may prolong local exposure to therapeutic cargo while simultaneously providing structural support to the injured tissue. Importantly, hydrogel‐based delivery can also be combined with electroconductive or neural tissue‐like scaffolds, potentially integrating the biological activities of exosomes with physical cues that support axonal growth and neural differentiation. Such composite systems have been investigated in SCI models. In a study conducted by Fan et al., bone marrow stem cell (BMSC)‐derived exosome‐loaded electroconductive hydrogels promoted microglial M2 polarization via the NF‐κB pathway, enhanced neuronal and oligodendrocyte differentiation, and stimulated axonal outgrowth through the PTEN/PI3K/AKT/mTOR pathway. In SCI mice, this composite system reduced microglial activation, increased neural stem cell (NSC) recruitment, promoted neuronal and axonal regeneration, and improved early functional recovery, highlighting its potential for SCI repair.^18^ Besides, other biomaterials, including 3D printing like 3D‐printed collagen‐chitosan scaffolds,^19^ nanomaterials like peptide amphiphile nanofibers,^20^ showed promising results in neural regeneration for SCI.^21^

Exosome engineering can further improve tissue targeting and cargo delivery. Surface modification with targeting peptides or ligands may enhance interaction with specific cell populations, while genetic or biochemical manipulation of donor cells can enrich therapeutic RNAs or proteins within exosomes. For example, the engineering of exosomes to carry circBDNF demonstrates how therapeutic cargo can be deliberately incorporated to activate specific neuroprotective pathways.^17^ Other strategies have incorporated targeting peptides like neuron targeting peptide (rabies viral glycoprotein [RVG]) and growth‐facilitating peptides (insulin‐like peptides [ILP] and intracellular sigma peptide [ISP]),^22^ antioxidant molecules like *Flos Sophorae Immaturus*,^23^ or ferroptosis‐regulating compounds to increase delivery efficiency and address specific secondary injury mechanisms.

Collectively, exosomes combine the biological advantages of endogenous intercellular communication with the engineering flexibility of a therapeutic delivery platform (Figure 1C). Their ability to transport diverse molecular cargoes, interact with multiple recipient cell types, and be modified for targeted or sustained delivery provides a strong rationale for their application in SCI.

## 3 IMPROVEMENTS IN MOTOR FUNCTION FOR SCI

Motor dysfunction represents the most significant manifestation of disability following SCI. Restoration of motor function depends on protecting residual neurons, promoting axonal growth across the injury site, and re‐establishing synaptic connections. Stem cell‐derived exosomes contribute to motor recovery through modulation of neuroinflammation, promotion of axonal regeneration and myelin repair, and inhibition of ferroptosis.

### 3.1 Neuroprotection and modulation of neuroinflammation

Neuroinflammation is a central driver of secondary SCI pathology. Several studies have demonstrated that stem cell‐derived exosomes exert neuroprotective effects by modulating inflammatory responses at the level of macrophage and microglial polarization. Exosomes from dental pulp stem cells suppress M1 macrophage polarization via inhibition of the ROS‐MAPK‐NFκB p65 signaling pathway, thereby alleviating secondary tissue damage.^24^ Similarly, exosomes derived from human umbilical cord MSCs (huc‐MSC‐Exos) reduce M1 microglial polarization both in vivo and in vitro, mediated through miR‐340‐5p modulation of the JAK/STAT3 pathway.^25^

Beyond polarization, exosomes also attenuate apoptosis and inflammatory infiltration. Shao et al. demonstrated that injection of BMSC exosomes overexpressing miR‐137 reduced tissue damage, inflammatory infiltration, and neuronal apoptosis in a rat SCI model, improving motor function and neuronal survival.^26^ Morishima et al. reported that intravenous injection of amniotic‐derived MSC exosomes at 24 h post‐injury reduced neutrophil infiltration and the formation of neutrophil extracellular traps at the lesion site through exosomal miR‐125a‐3p, with motor function improvements sustained over 28 days.^27^

Stem cell‐derived exosomes can alleviate programmed cell death to exert neuroprotection. Ferroptosis is an iron‐dependent form of regulated cell death characterized by excessive lipid peroxidation and contributes substantially to secondary neuronal and oligodendrocyte injury after SCI. Increasing evidence indicates that stem cell‐derived exosomes suppress ferroptosis through multiple molecular pathways. MSC‐derived exosomes inhibit microglial ferroptosis through the Nrf2/GCH1/BH4 axis, promoting neural functional recovery.^28^ BMSC‐derived exosomes (BMSC‐Exos) also reduce iron accumulation, lipid peroxidation, and ROS production through inhibition of the interleukin (IL)‐17 pathway, thereby suppressing ferroptosis and improving functional recovery.^29^ Mechanistically engineered exosomes provide additional ferroptosis‐targeting strategies. NSC‐derived exosomes delivering miR‐218a‐5p inhibit Bmi1 and promote Mettl3 degradation, subsequently reducing Alox12 mRNA m6A methylation and Alox12 expression through YTHDF2, thereby suppressing ferroptosis.^30^ BMSC‐derived exosomal AKAP12 similarly inhibits STAT3 and ERK signaling, reducing iron transport and lipid peroxidation and protecting neurons from ferroptosis.^31^ In another study, miR‐219‐5p‐enriched BMSC exosomes reduced Fe^2+^ and malondialdehyde (MDA) levels while preserving glutathione (GSH) and superoxide dismutase (SOD), indicating suppression of ferroptosis. Exosomal miR‐219‐5p targeted ubiquitin‐conjugating enzyme E2 Z (UBE2Z) and activated the nuclear factor erythroid 2‐related Factor 2 (NRF2) pathway, whereas UBE2Z overexpression or miR‐219‐5p depletion weakened the protective effects, supporting a UBE2Z/NRF2‐dependent mechanism.^32^ Other forms of programmed cell death are also targeted by stem cell‐derived exosomes. BMSC‐Exos carrying circ_003564 suppresses inflammasome‐mediated pyroptosis by reducing cleaved caspase‐1, GSDMD, NLRP3, IL‐1β, and IL‐18, thereby limiting tissue damage and improving functional recovery in both rat SCI models and H_2_O_2_‐treated primary neurons.^33^

Collectively, these findings indicate that stem cell‐derived exosomes mitigate secondary injury through coordinated regulation of inflammatory cell polarization, reduction of apoptosis, and inhibition of ferroptosis.

### 3.2 Promotion of axonal regeneration and myelin repair

Axonal disruption is a direct cause of motor function loss following SCI. Stem cell‐derived exosomes have been shown to promote axonal regrowth and myelin repair through delivery of neurotrophic factors and regulatory RNAs that activate key regenerative signaling cascades.

Exosomes carrying specific miRNAs can directly enhance axonal regrowth. Intravenous injection of miR‐133b‐containing MSC‐derived exosomes reduced lesion volume, preserved neurons, and promoted axonal regeneration, improving hindlimb functional recovery in a rat SCI model.^34^ Direct administration of exosome‐educated macrophages derived from BMSCs likewise improved neurological recovery by promoting angiogenesis and axonal growth.^35^ Engineering approaches have further improved exosome targeting and efficacy. Li et al. subsequently demonstrated that intravenous administration of BMSC‐Exos overexpressing miR‐24‐3p reduced apoptosis and inflammation, promoted axonal growth, and alleviated motor dysfunction by targeting MAPK9 to inhibit the JNK/c‐Jun/c‐Fos pathway.^36^ Transplantation of exosomes derived from BMSC sheets overexpressing nerve growth factor further accelerated neural functional recovery by promoting NSC differentiation into neurons and facilitating axonal regeneration.^37^ Similarly, Xie et al. demonstrated that local delivery of exosomes from an epidermal growth factor receptor‐positive NSC subpopulation enhanced axonal regeneration and neurological recovery.^38^

Biomaterial combination strategies have demonstrated complementary benefits. Fan et al. reported that the combined use of BMSC‐Exos and conductive hydrogels reduced early inflammation, enhanced NSC recruitment, and promoted both neuronal differentiation and axonal regeneration.^18^ Another study showed that intravenous injection of a MSC‐membrane‐coated mesoporous silica nanoparticle complex carrying miR‐124‐3p specifically crossed the compromised blood and spinal cord barrier, accumulated at the injury site, and facilitated axonal regeneration and functional restoration.^39^

Together, these studies highlight the capacity of stem cell‐derived exosomes to promote axonal regeneration and myelin repair through miRNA delivery, advanced engineering strategies, and biomaterial synergy.

## 4 PATHOPHYSIOLOGY OF SENSORY DYSFUNCTION AND NEUROPATHIC PAIN AFTER SCI

Beyond motor dysfunction, SCI produces complex and persistent disturbances in sensory processing that extend beyond the initial mechanical damage. Sensory dysfunction may manifest as impaired touch, proprioception, vibration, and temperature perception, whereas maladaptive changes within injured and spared neural circuits can give rise to neuropathic pain, including spontaneous pain, mechanical allodynia, and thermal hyperalgesia. The pathophysiology of these conditions involves not only direct disruption of ascending sensory tracts but also secondary neuroinflammation, glial activation, neuronal hyperexcitability, blood‐spinal cord barrier (BSCB) disruption, oxidative stress, and maladaptive synaptic remodeling. Understanding the interactions among these pathological processes is essential for identifying therapeutic strategies capable of simultaneously promoting sensory restoration and suppressing neuropathic pain.

### 4.1 Disruption of ascending sensory pathways and sensory dysfunction

The anatomical disruption of ascending sensory pathways is a primary determinant of sensory impairment after SCI. Somatosensory information is transmitted to supraspinal centers through several anatomically and functionally distinct pathways. The dorsal column–medial lemniscus system primarily conveys fine touch, vibration, and proprioception, whereas the anterolateral system, including the spinothalamic tract, is predominantly involved in the transmission of pain and temperature.^40^ Following SCI, axonal transection, demyelination, neuronal loss, and disruption of synaptic connectivity within these pathways can interrupt the transmission of sensory information from peripheral receptors to the brain.

The extent and pattern of sensory impairment depend on the anatomical level, severity, and completeness of the injury. Lesions involving the dorsal columns may markedly impair proprioception and discriminative touch, whereas damage to the anterolateral pathways can compromise nociceptive and thermal perception, with dorsal root ganglia (DRG) and axons affected.^41^ Importantly, the sensory consequences of SCI cannot be explained solely by the number of axons physically destroyed at the time of injury. Secondary injury processes progressively expand tissue damage and compromise initially spared neural structures. Edema, ischemia, excitotoxicity, demyelination, and inflammatory responses can further impair axonal conduction and disrupt the integrity of neural circuits surrounding the lesion.

Spontaneous axonal regeneration within the adult central nervous system (CNS) is limited, and the injured spinal cord develops a non‐permissive environment characterized by glial scar formation, extracellular matrix remodeling, and persistent inflammation.^42^ Consequently, surviving axons may fail to establish appropriate connections across the lesion. In addition, impaired remyelination can reduce conduction efficiency even when axonal continuity is partially preserved. These structural and functional abnormalities provide an important basis for persistent sensory deficits after SCI and highlight the need for therapeutic strategies that promote axonal regeneration, remyelination, synaptic reconstruction, and restoration of functional sensory circuits. For example, in a rat spinal cord dorsal column lesion (SDCL) model, miR‐20a expression decreased progressively after SDCL. Modulation of miR‐20a enhanced axonal outgrowth of primary dorsal root ganglion neurons in vitro and regulated the PDZ‐RhoGEF/RhoA/GAP43 signaling axis. In vivo, miR‐20a modulation promoted recovery of ascending sensory function and altered the miR‐20a/PDZ‐RhoGEF/RhoA/GAP43 pathway.^43^ These findings suggest that regenerative regimen like exosomes targeting the miR‐20a/PDZ‐RhoGEF/RhoA/GAP43 axis may facilitate sensory axonal regeneration and functional recovery after SCI.

### 4.2 Neuroinflammation and glial activation in neuropathic pain

In contrast to sensory loss caused primarily by structural interruption of ascending pathways, neuropathic pain after SCI is strongly associated with persistent neuroinflammation and maladaptive activation of spinal glial cells.^44^ Microglia and astrocytes become activated following SCI in response to tissue damage, cellular debris, disrupted extracellular homeostasis, and inflammatory signals. Although glial activation initially contributes to tissue repair and host defense, prolonged activation can become detrimental and promote chronic pain.^45^

Activated microglia release a variety of inflammatory mediators, including tumor necrosis factor‐α (TNF‐α), interleukin‐1β (IL‐1β), and other cytokines and chemokines that enhance neuronal excitability and alter synaptic transmission within the dorsal horn. Toll‐like receptor (TLR)‐dependent signaling, particularly the TLR2/TLR4–MyD88–NF‐κB axis, represents an important molecular pathway linking tissue injury to sustained microglial inflammatory activation.^7^ Persistent activation of this pathway promotes the production of proinflammatory mediators and contributes to the maintenance of neuropathic pain.

Astrocytes also undergo prolonged reactive changes after SCI. Reactive astrocytes can alter extracellular ion and neurotransmitter homeostasis, release inflammatory mediators, and contribute to pathological synaptic remodeling. At the lesion site, excessive astrocytic proliferation and extracellular matrix deposition contribute to glial scar formation, which restricts axonal regeneration. However, the role of astrocytes in pain is not restricted to the lesion itself. Reactive astrocytes within pain‐processing regions can participate in the maintenance of abnormal neuronal excitability and central sensitization. Besides, SCI increased astrocytic exosomal lncRNA49rik, which promoted microglial activation, neuroinflammation, oxidative stress, and neuropathic pain. Mechanistically, lncRNA49rik negatively regulated miR‐10a‐5p, while miR‐10a‐5p overexpression alleviated mechanical and thermal hypersensitivity and suppressed inflammatory responses. Further analyses indicated that the lncRNA49rik/miR‐10a‐5p axis modulated microglial phenotypes through the MAPK/PI3K/AKT/mTOR pathway. These findings also suggest that astrocyte‐derived exosomal lncRNA49rik contributes to SCI‐induced neuropathic pain by promoting microglial activation and inflammation through miR‐10a‐5p‐dependent signaling.^46^

Abnormal lncRNAs influence the activation of inflammatory cytokines, microRNAs, and other related molecules, which are crucial to the development of chronic pain.^47^ In a rat SCI model, the role of the lncRNA nuclear paraspeckle assembly transcript 1 (Neat1) in primary sensory neurons was investigated using transcriptomic and molecular analyses. Neat1 was highly expressed in the DRG and upregulated following nerve injury. Neat1 overexpression in sensory neurons exacerbated mechanical and thermal hypersensitivity, whereas its knockdown alleviated neuropathic pain. Transcriptomic analysis identified inflammatory responses as the major biological process associated with Neat1‐regulated genes, and Neat1 knockdown suppressed the upregulation of multiple proinflammatory genes in the DRG. Specifically, Neat1 stabilized interacting proinflammatory mRNAs and promoted spinal inflammatory responses, thereby facilitating peripheral neuropathic pain.^48^

Altogether, neuroinflammation establishes a pathological interface between tissue injury and sensory dysfunction. On the one hand, excessive inflammation damages surviving neurons, oligodendrocytes, and axons, thereby worsening sensory loss. On the other hand, persistent glial activation promotes abnormal nociceptive signaling and contributes to neuropathic pain. This dual role makes neuroinflammation an important therapeutic target for interventions aimed at simultaneously restoring sensory function and alleviating pain.

### 4.3 Central sensitization and aberrant neuronal excitability

Persistent neuroinflammation and structural disruption of sensory circuits can induce profound changes in neuronal excitability after SCI. Central sensitization refers to an abnormal increase in the responsiveness of neurons within the CNS to sensory inputs, resulting in amplification of otherwise innocuous or weak stimuli, with enhanced temporal summation of tonic heat pain and diminished habituation of pain‐induced sympathetic measures.^49^, ^50^ In the spinal dorsal horn, increased excitatory neurotransmission, altered inhibitory control, and inflammatory signaling collectively contribute to the development and maintenance of central sensitization.

Following SCI, loss of descending inhibitory control from supraspinal centers may further increase the excitability of spinal nociceptive networks. At the same time, persistent activation of primary afferent neurons and dorsal horn neurons can promote activity‐dependent synaptic strengthening. Changes in glutamatergic transmission, ion channel function, and intracellular signaling pathways can further increase neuronal responsiveness. For example, dopamine D1 receptor (D1DR) and D2 receptor (D2DR) form a complex in the rat spinal cord and, in turn, couple with the Gαq protein to increase neuronal excitability via PKC γ/CaMKII/MAPK/CREB signaling in the spinal cords of injured rats.^51^ In a rat contusive SCI model, sodium currents and excitability were examined in small DRG neurons. SCI markedly increased Nav1.8‐mediated transient and resurgent sodium currents and increased the proportion of neurons exhibiting tetrodotoxin‐resistant resurgent currents, resulting in enhanced neuronal excitability. Treatment with ZL0177, a peptidomimetic that disrupts fibroblast growth factor homologous factor (FHF) binding to the C‐terminal region of sodium channels, reduced Nav1.8/Nav1.9 currents, decreased the proportion of neurons with Nav1.8 resurgent currents, and attenuated SCI‐induced DRG neuronal hyperexcitability, suggesting that dysregulated Nav1.8 activity contributes to SCI‐associated neuropathic pain and that targeting FHF modulation of Nav1.8/Nav1.9 may represent a potential therapeutic strategy.^52^ These alterations also explain why patients can experience severe pain even when sensory perception is simultaneously reduced.

### 4.4 BSCB disruption and secondary injury

The BSCB is a specialized neurovascular interface that maintains spinal cord homeostasis by restricting the uncontrolled exchange of circulating molecules and immune cells with neural tissue.^53^ Its integrity depends on coordinated interactions among endothelial cells, pericytes, basement membrane components, and surrounding glial cells. SCI rapidly disrupts this neurovascular unit, resulting in increased vascular permeability and leakage of plasma proteins and immune cells into the injured spinal cord,^54^ creating a neuroinflammation environment for neuropathic pain.

BSCB disruption contributes to secondary injury through several interconnected mechanisms. Increased vascular permeability promotes tissue edema and elevates local pressure, while infiltration of peripheral immune cells intensifies inflammatory responses. Matrix metalloproteinases (MMPs) can degrade extracellular matrix components and endothelial junction proteins, further compromising barrier integrity.^55^ In parallel, endothelial dysfunction and pericyte loss can destabilize the microvasculature and impair tissue perfusion.^56^

The consequences of BSCB disruption extend beyond the lesion itself. Persistent vascular leakage can sustain neuroinflammation, increase oxidative stress, and expose neurons and glial cells to potentially damaging circulating factors.^57^ Conversely, inflammatory mediators and ROS can further impair endothelial and pericyte function, creating a positive feedback loop that prolongs barrier dysfunction. Therefore, BSCB disruption represents an important link between vascular injury, neuroinflammation, neuronal damage, and impaired neural regeneration.

From a therapeutic perspective, restoration of BSCB integrity may protect surviving neural tissue while simultaneously creating a more permissive environment for axonal regeneration and sensory circuit reconstruction. This provides a mechanistic rationale for targeting the neurovascular unit as part of comprehensive treatment strategies for SCI‐associated sensory dysfunction and neuropathic pain.

Taken together, the pathophysiology of SCI‐associated sensory dysfunction and neuropathic pain can be conceptualized as a dynamic interaction among structural disruption of sensory pathways, neuroinflammation and glial activation, neuronal hyperexcitability and central sensitization, and BSCB dysfunction (Figure 2). These interconnected mechanisms provide multiple therapeutic entry points and establish the mechanistic foundation for the regenerative, analgesic, and barrier‐protective effects of stem cell‐derived exosomes discussed in the following section.

**Figure 2:**
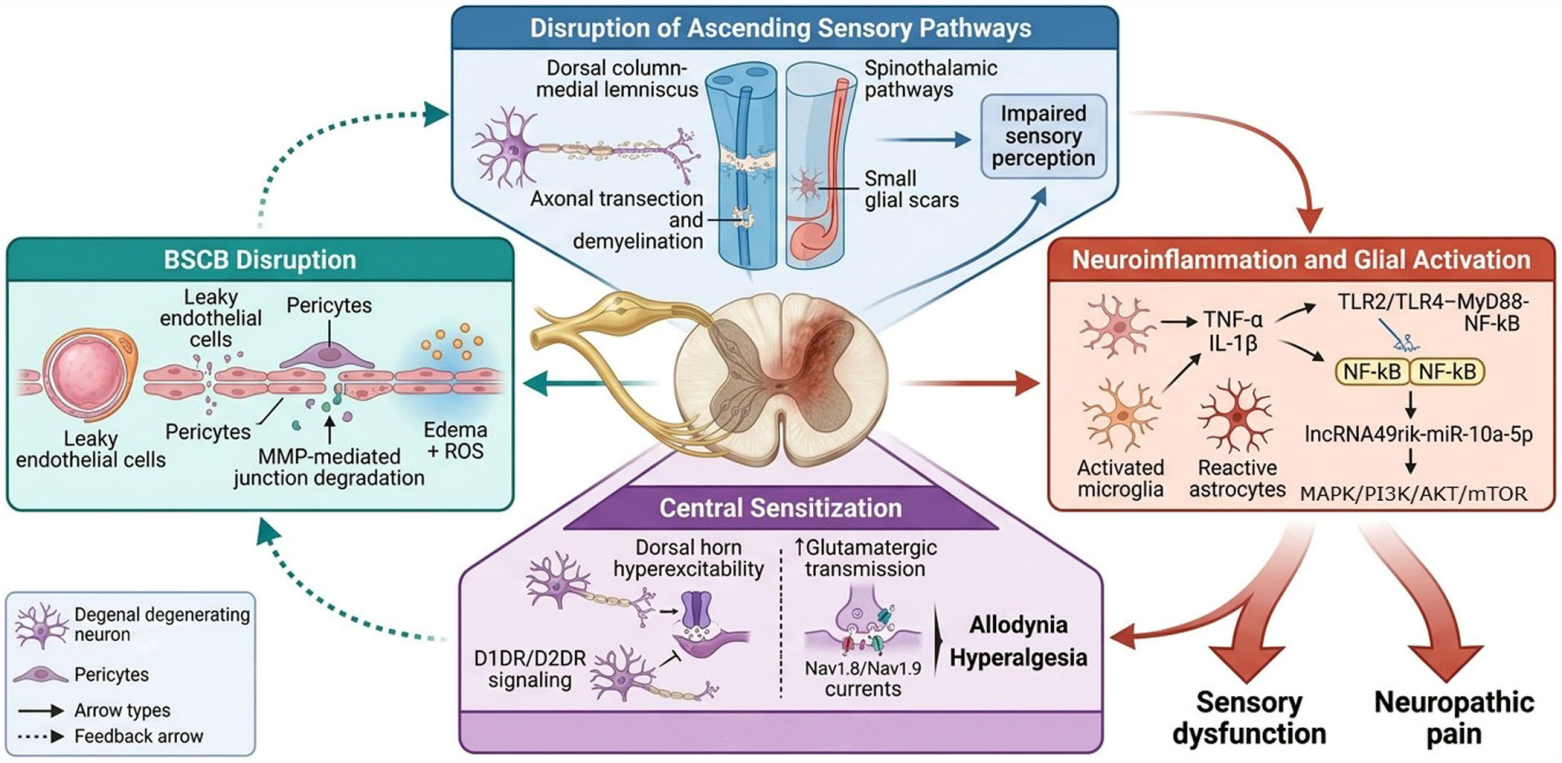
The pathophysiology of SCI‐associated sensory dysfunction and neuropathic pain. The underlying mechanisms are multifactorial and involve structural disruption of sensory pathways, neuroinflammation with glial activation, neuronal hyperexcitability and central sensitization, as well as BSCB dysfunction. BSCB, blood–spinal cord barrier; ROS, reactive oxygen species.

## 5 STEM CELL‐DERIVED EXOSOMES FOR SENSORY RESTORATION AND NEUROPATHIC PAIN RELIEF

Sensory dysfunction following SCI encompasses loss of touch, proprioception, and temperature discrimination, and is frequently accompanied by debilitating neuropathic pain. These deficits profoundly impact quality of life and present distinct therapeutic challenges compared to motor recovery. Stem cell‐derived exosomes have demonstrated emerging potential for the restoration of sensory function through three principal mechanisms: restoration of sensory function, alleviation of neuropathic pain and maintenance of BSCB integrity.

### 5.1 Restoration of sensory function

Damage to ascending sensory tracts, including the dorsal column and spinothalamic pathways, is a major anatomical basis for sensory impairment following SCI. Axonal disruption and demyelination can severely compromise the transmission of tactile, proprioceptive, thermal, and nociceptive information to supraspinal centers. Therefore, effective sensory restoration requires not only neuronal survival but also reconstruction of functional connections across the injured spinal cord. Stem cell‐derived exosomes may contribute to this process through their neuroprotective and regenerative activities. Exosomal proteins, growth factors, and non‐coding RNAs can regulate pathways involved in axonal growth, cytoskeletal remodeling, neuronal differentiation, and myelination.

A major mechanism underlying the potential sensory‐restorative effects of stem cell‐derived exosomes is the promotion of axonal regeneration. A recent study used single‐cell RNA sequencing and flow cytometry to identify a CD271^+^CD56^+^ subpopulation of BMSCs with enhanced axon‐regenerative properties. Exosomes derived from these cells were incorporated into a hydrogel to achieve sustained delivery after SCI. In vitro, CD271^+^CD56^+^ BMSC‐Exos markedly promoted DRG axonal extension and branching. In vivo, implantation of the exosome‐loaded hydrogel increased neurofilament and synaptophysin expression and improved neurological recovery. Mechanistically, these effects were associated with activation of the miR‐431‐3p/RGMA axis, indicating that exosomal miR‐431‐3p may contribute to axonal growth and remodeling of neuronal connections after injury.^58^ Although the study primarily evaluated structural and overall neurological recovery rather than sensory‐specific behavioral outcomes, enhanced axonal regeneration and synaptic remodeling may provide an important anatomical basis for restoring ascending sensory transmission.

Engineering the molecular cargo of BMSC‐Exos may further enhance their regenerative capacity. In a SDCL rat model, BMSC‐Exos loaded with a miR‐17‐5p inhibitor were investigated as a strategy to improve neural repair. Transcriptomic and miRNomic analyses implicated the JAK/STAT signaling pathway in exosome‐mediated neuroregeneration. Compared with miR‐17‐5p inhibition or unmodified BMSC‐Exos alone, miR‐17‐5p inhibitor‐loaded exosomes produced greater DRG axonal outgrowth and improved sensory conduction after SDCL. These effects were associated with activation of the IL‐11/GP130/STAT3/GAP‐43 axis, linking exosomal cargo modification to axonal growth and functional sensory transmission.^9^ It highlights the possibility that engineering‐specific non‐coding RNA cargoes can increase the capacity of stem cell‐derived exosomes to repair damaged sensory pathways rather than relying solely on the endogenous properties of naturally derived exosomes.

The regenerative effects of stem cell‐derived exosomes may also be enhanced by combining exosome delivery with biomaterial scaffolds that provide structural and biophysical support. BMSC‐Exos incorporated into a neural tissue‐like electroconductive hydrogel promoted M2 microglial polarization through NF‐κB pathway modulation, enhanced neuronal and oligodendrocyte differentiation of NSCs, and suppressed astrocytic differentiation. The exosome‐loaded hydrogel also increased axonal outgrowth through the PTEN/PI3K/AKT/mTOR pathway. In vivo, this composite system reduced CD68^+^ microglial accumulation, increased local NSC recruitment, and promoted neuronal and axonal regeneration, ultimately improving early neurological recovery after SCI.^18^ These findings suggest that exosomes can function not only as biological carriers but also as components of multifunctional regenerative systems in which immunomodulation, neural differentiation, and axonal growth are coordinated within the injured spinal cord. Consistent with this concept, M2 microglia‐derived exosomes (M2‐Exos) have been incorporated into electroconductive hydrogels to combine the immunoregulatory properties of exosomes with the physical and electrical characteristics of biomaterial scaffolds. In vitro, M2‐Exos‐loaded hydrogels promoted NSC proliferation and DRG axonal growth, enhanced M2 microglial polarization, and attenuated the early inflammatory response. In SCI rats, the composite hydrogel further enhanced neuronal and axonal regeneration and improved neurological functional recovery.^59^ Because excessive and persistent inflammation can inhibit axonal growth and disrupt neural circuit reconstruction, modulation of the inflammatory microenvironment may indirectly facilitate the restoration of sensory pathways. Thus, exosome‐based immunomodulation and axonal regeneration should be considered interconnected rather than independent mechanisms of sensory recovery.

Beyond direct effects on neurons and inflammatory cells, stem cell‐derived exosomes may support axonal regeneration by reshaping the metabolic environment of the injured spinal cord. Olfactory mucosa MSC‐derived exosomes enriched in the lncRNA RMRP (OM‐MSC‐Exos‐RMRP) were shown to enhance astrocytic glycolysis, glucose consumption, and lactate production, accompanied by increased axonal regeneration after SCI. Depletion of RMRP abolished these effects, indicating that exosomal RMRP was a critical mediator. Mechanistically, RMRP interacted with WTAP and reduced WTAP‐mediated m6A modification of p53 mRNA, thereby promoting p53 destabilization and inducing metabolic reprogramming of astrocytes. In vivo, OM‐MSC‐Exos enhanced glycolysis, axonal regeneration, and neurological recovery, supporting a model in which exosome‐mediated metabolic remodeling of glial cells creates a more favorable environment for neural repair.^60^ Although the reported functional outcomes were primarily related to overall neurological recovery rather than sensory‐specific behaviors, restoration of a metabolically supportive glial niche may facilitate axonal maintenance and regeneration within ascending sensory pathways.

Collectively, current evidence indicates that stem cell‐derived exosomes may contribute to sensory restoration after SCI through a multifaceted regenerative network involving direct promotion of axonal growth, synaptic remodeling, neural lineage regulation, inflammatory microenvironmental modulation, and metabolic reprogramming of glial cells. Importantly, these mechanisms appear to be interconnected: enhanced axonal regeneration requires a permissive inflammatory and metabolic environment, while appropriate neural differentiation and remyelination are necessary for newly regenerated axons to establish functional sensory circuits. Nevertheless, most existing studies have evaluated axonal regeneration, histological markers, or general neurological scores, whereas direct assessments of tactile, proprioceptive, thermal, and nociceptive function remain relatively limited. Future studies should therefore incorporate sensory‐specific behavioral paradigms, electrophysiological assessment of ascending pathways, and circuit‐level analyses to determine whether the structural repair induced by stem cell‐derived exosomes ultimately translates into meaningful restoration of sensory perception.

### 5.2 Alleviation of neuropathic pain

Spontaneous pain and hyperalgesia following SCI are typically associated with glial cell hyperactivation, central sensitization, and aberrant dorsal horn signaling. Stem cell‐derived exosomes have shown consistent efficacy in attenuating these pain‐related mechanisms across multiple experimental models.

Microglial activation and TLR‐mediated inflammatory signaling represent important mechanisms underlying neuropathic pain after SCI. In a mouse model of SCI‐induced neuropathic pain (SCI‐NP), genetic deficiency of TLR4 reduced pain hypersensitivity, microglial activation, and activity of the TLR4/MyD88/NF‐κB signaling cascade. Consistent with these findings, local administration of BMSC‐Exos significantly alleviated mechanical hypersensitivity and suppressed microglial activation, accompanied by downregulation of TLR4/MyD88/NF‐κB signaling.^10^ These findings suggest that BMSC‐Exos may exert analgesic effects by suppressing the inflammatory signaling cascade that links spinal microglial activation to enhanced nociceptive transmission. Importantly, this mechanism provides a direct connection between exosome‐mediated immunomodulation and the attenuation of SCI‐associated pain hypersensitivity.

A similar regulatory mechanism has been observed with huc‐MSC‐Exos, although the implicated TLR pathway differs. In vitro experiments demonstrated that huc‐MSC‐Exos inhibited microglial activation and suppressed TLR2/MyD88/NF‐κB signaling. Proteomic analysis identified radical S‐adenosyl methionine domain‐containing 2 (Rsad2) as a potential downstream target, while Rsad2 knockdown reproduced the inhibitory effects on microglial activation and inflammatory signaling. In vivo, intrathecal administration of huc‐MSC‐Exos reduced mechanical allodynia in a chronic constriction injury model and suppressed Rsad2 expression and TLR2/MyD88/NF‐κB signaling in spinal microglia.^61^ Together with the findings from TLR4 inhibition, these observations indicate that stem cell‐derived exosomes may converge on the MyD88/NF‐κB inflammatory axis through distinct upstream TLR‐dependent mechanisms, thereby limiting microglia‐driven neuroinflammation and pathological pain sensitization.

The analgesic effects of stem cell‐derived exosomes may also be achieved through simultaneous regulation of the injured neural microenvironment. In a T9 hemisection rat model of SCI, huc‐MSC‐Exos were delivered locally using an exosome‐loaded Gelfoam scaffold. Tracing studies demonstrated that the exosomes were internalized by neurons, microglia, astrocytes, and oligodendrocyte‐lineage cells surrounding the lesion. Treatment improved locomotor and gait performance while alleviating neuropathic pain and enhancing axonal regeneration, remyelination, and synaptic formation, as reflected by increased neurofilament‐200 (NF200), growth‐associated protein 43 (GAP43), synaptophysin, and postsynaptic density 95 (PSD95) expression.^62^ In parallel, huc‐MSC‐Exosreduced astroglial scarring, microglial activation, inflammation, and apoptosis, as indicated by decreased glial fibrillary acidic protein (GFAP), chondroitin sulfate proteoglycan (CSPG), ionized calcium binding adaptor molecule 1 (Iba1), inducible nitric oxide synthase (iNOS), Bax, and p75NTR expression.^62^ These findings suggest that the analgesic effects of exosomes may not result from suppression of a single pain‐related pathway but rather from coordinated restoration of the injured neural environment. By reducing glial reactivity and inflammation while promoting axonal regeneration, remyelination, and synaptic remodeling, stem cell‐derived exosomes may simultaneously reduce pathological pain signaling and facilitate neural repair.

The delivery platform itself may further influence the therapeutic effects of stem cell‐derived exosomes. In another SCI study, adipose‐derived MSC exosomes (AD‐MSC‐Exos) were incorporated into collagen or fibrin hydrogels. Both delivery systems improved neurological recovery and reduced SCI‐associated tissue degeneration.^63^ These changes were associated with enhanced spinal cord tissue repair and reduced central neuropathic pain. Although the molecular mechanisms responsible for the analgesic effects were not fully established, these findings support the concept that biomaterial‐assisted exosome delivery can prolong local exposure and integrate pain modulation with structural tissue regeneration.

Evidence from peripheral nerve injury models further supports the analgesic and neurotrophic properties of stem cell‐derived exosomes. In a rat L5/L6 spinal nerve ligation model, intrathecal administration of huc‐MSC‐Exos alleviated both mechanical and thermal hypersensitivity during early and established stages of neuropathic pain.^64^ Fluorescent tracing demonstrated exosome accumulation in the ipsilateral dorsal horn, DRG, peripheral axons, and sensory neurons, indicating that exosomes can reach multiple anatomical sites involved in pain processing. Exosome treatment reduced neuronal and glial activation, as reflected by decreased c‐Fos, GFAP, and Iba1 expression, while suppressing TNF‐α and IL‐1β and increasing IL‐10, BDNF, and glial cell line‐derived neurotrophic factor (GDNF) levels.^64^ These findings suggest that stem cell‐derived exosomes can attenuate neuropathic pain through a combination of anti‐inflammatory and neurotrophic effects, simultaneously reducing pathological neuronal and glial activation while enhancing endogenous mechanisms of neural repair.

Sustained local delivery may further enhance these effects. In the same spinal nerve ligation model, huc‐MSC‐Exos were incorporated into an alginate scaffold and wrapped around the injured nerves. The exosome‐loaded scaffold promoted neurite outgrowth in vitro and protected neuronal and non‐neuronal cells from injury. In vivo, treatment significantly reduced mechanical allodynia and thermal hyperalgesia, accompanied by decreased c‐Fos, GFAP, Iba1, TNF‐α, and IL‐1β expression and increased IL‐10 and GDNF levels in the affected DRG.^65^ Increased myelin basic protein and IL‐10 expression in injured axons further suggested enhanced remyelination and resolution of the local inflammatory response. These results demonstrate that scaffold‐based exosome delivery can provide sustained local exposure while simultaneously exerting antinociceptive, anti‐inflammatory, and neurotrophic effects. Such approaches may be particularly relevant for SCI, where prolonged retention of therapeutic cargo within the lesion or surrounding sensory circuitry remains a major challenge.

In addition to suppressing classical inflammatory signaling, stem cell‐derived exosomes may alleviate pain by regulating inflammatory cell death. In a complete Freund's adjuvant‐induced inflammatory pain model, intrathecal huc‐MSC‐Exos reduced mechanical allodynia and thermal hyperalgesia by enhancing autophagy and suppressing NLRP3 inflammasome activation and pyroptosis in spinal microglia. Mechanistically, exosomal miR‐146a‐5p promoted autophagy and inhibited microglial pyroptosis by targeting TNF receptor‐associated factor 6 (TRAF6), whereas pharmacological inhibition of autophagy attenuated the protective effects of huc‐MSC‐Exos.^66^ Although this study was performed in an inflammatory pain model rather than SCI, it provides mechanistic evidence that exosomal microRNAs can regulate the balance between protective autophagy and inflammatory pyroptosis in spinal microglia. Given the important contribution of NLRP3 activation and microglial inflammatory signaling to chronic pain, this mechanism may represent an additional pathway through which stem cell‐derived exosomes could modulate persistent neuropathic pain after SCI.

Taken together, the available evidence indicates that stem cell‐derived exosomes alleviate neuropathic pain through coordinated regulation of neuroinflammation, glial activation, neuronal hyperexcitability, and tissue repair. The TLR2/TLR4–MyD88–NF‐κB axis emerges as an important inflammatory pathway targeted by MSC‐derived exosomes, whereas regulation of Rsad2, miR‐146a‐5p/TRAF6 signaling, autophagy, and NLRP3‐associated pyroptosis further expands the range of molecular mechanisms involved. At the cellular level, suppression of activated microglia and astrocytes, reduction of proinflammatory cytokines, enhancement of neurotrophic signaling, and promotion of remyelination may collectively reduce pathological nociceptive amplification. Notably, the analgesic effects of stem cell‐derived exosomes may therefore complement their regenerative actions: restoration of axonal integrity and synaptic organization may reduce abnormal sensory signaling, while suppression of neuroinflammation may create a permissive environment for neural repair.

Nevertheless, an important limitation of the current evidence is that studies using SCI models remain relatively fewer than those using peripheral nerve injury or inflammatory pain models. Moreover, improvements in pain‐related behavioral tests do not necessarily establish normalization of sensory circuit activity, and the relationship between exosome‐mediated tissue repair and long‐term resolution of central sensitization remains incompletely understood. Future studies should therefore combine mechanical and thermal pain assays with electrophysiological recordings, dorsal horn circuit analysis, and longitudinal assessment of sensory pathway integrity. Such approaches will help determine whether stem cell‐derived exosomes merely suppress pain‐related hypersensitivity or can induce durable remodeling of the pathological neural circuits responsible for chronic neuropathic pain after SCI.

### 5.3 Maintenance of BSCB integrity

The BSCB plays a critical role in maintaining the homeostatic spinal cord microenvironment, and its disruption following SCI exacerbates secondary injury by permitting immune cell infiltration and edema. Preservation or restoration of BSCB integrity is therefore a key therapeutic objective for both motor and sensory recovery.

Accumulating evidence indicates that stem cell‐derived exosomes can protect the BSCB by acting on multiple components of the neurovascular unit, including endothelial cells, pericytes, and surrounding glial cells. One important mechanism involves direct preservation of endothelial barrier function. In a rat SCI model and an in vitro BSCB injury model using human brain microvascular endothelial cells, BMSC‐Exos reduced BSCB permeability and promoted tissue repair. Mechanistically, exosomal tissue inhibitor of metalloproteinases 2 (TIMP2) was identified as an important mediator of this effect. BMSC‐Exos suppressed MMP activity and preserved endothelial junction proteins, whereas TIMP2 knockdown markedly attenuated the barrier‐protective effects of the exosomes.^67^ These findings suggest that exosomal cargo can directly regulate extracellular matrix remodeling and endothelial junction stability, thereby limiting vascular leakage after SCI.

Pericytes, which provide structural and functional support to spinal microvessels, represent another important target of BMSC‐Exos. In a T10 impact‐induced SCI rat model, intravenous administration of BMSC‐Exos shortly after injury reduced neuronal death, myelin loss, edema, and BSCB leakage, while increasing pericyte and endothelial coverage of the vascular wall and improving locomotor recovery. In vitro, BMSC‐Exos protected pericytes from cytokine‐ and lipopolysaccharide (LPS)/adenosine triphosphate (ATP)‐induced pyroptosis and enhanced their survival. These effects were associated with reduced caspase‐1 expression and IL‐1β release, indicating that suppression of pericyte pyroptosis contributes to BSCB preservation. Because loss or dysfunction of pericytes can destabilize the neurovascular unit and increase vascular permeability, protection of pericytes may represent a complementary mechanism through which BMSC‐Exos maintain barrier integrity after SCI.^68^

The importance of exosomal regulation of the vascular compartment is further illustrated by studies examining the pathological effects of macrophage‐derived exosomes. Following SCI, BSCB disruption facilitates infiltration of peripheral macrophages into the lesion, where M1‐polarized macrophages can further aggravate vascular dysfunction. M1 bone marrow‐derived macrophage exosomes promoted endothelial‐to‐mesenchymal transition (EndoMT), mitochondrial dysfunction, and ROS accumulation in endothelial cells, thereby exacerbating BSCB disruption. Mechanistically, exosomal miR‐155 was transferred to endothelial cells and targeted suppressor of cytokine signaling 6 (SOCS6), reducing SOCS6‐mediated p65 ubiquitination and degradation and consequently activating NF‐κB signaling. Rescue experiments confirmed that the miR‐155/SOCS6/p65 axis mediated endothelial dysfunction and mitochondrial abnormalities.^69^ Although these exosomes are not therapeutic stem cell‐derived exosomes, this study provides important mechanistic evidence that exosomal communication between inflammatory cells and endothelial cells can critically determine BSCB integrity. It also highlights the importance of selecting an appropriate cellular source when developing exosome‐based therapies, because exosomes released under inflammatory conditions may instead promote vascular injury.

Beyond preserving existing endothelial junctions, stem cell‐derived exosomes may promote vascular regeneration and remodeling after SCI. Human umbilical cord MSCs induced toward an endothelial‐like phenotype produce exosomes with enhanced vascular regulatory properties. These endothelial‐like huc‐MSC‐Exos (E‐UCMSC‐Exos) promoted endothelial cell migration and improved BSCB stability in vitro. To enhance vascular targeting, the exosomes were further modified with arginine‐glycine‐aspartic acid (RGD) peptides. In SCI mice, RGD‐E‐UCMSC‐Exos preferentially accumulated in neovascular endothelial cells, promoted angiogenesis, reinforced BSCB integrity, and improved neurological recovery. Proteomic analysis identified MMP2, FLT1, TIMP1, GAS6, CTHRC1, and NEO1 as potential mediators of their angiogenic and barrier‐stabilizing effects.^70^ These findings suggest that exosome‐based therapy may simultaneously promote vascular regeneration and restore barrier function, rather than merely preventing vascular leakage.

A related strategy involved engineering CD146^+^CD271^+^ huc‐MSC‐Exos with RGD peptides to specifically target neovascular endothelial cells. Intranasal administration of RGD‐CD146^+^CD271^+^ huc‐MSC‐Exos enhanced exosome accumulation at the injured spinal cord, reduced BSCB permeability, and increased the expression of tight junction proteins. In oxygen‐glucose deprivation‐injured endothelial cells, these engineered exosomes reduced endothelial permeability and restored junctional integrity. Mechanistically, the protective effects were associated with the regulation of the miR‐501‐5p/myosin light chain kinase axis.^38^ Together with the findings from RGD‐E‐UCMSC‐Exos, these results demonstrate the potential of ligand‐engineered exosomes to overcome the limited targeting efficiency of conventional exosome therapy and preferentially deliver therapeutic cargo to the injured vasculature.

The barrier‐protective effects of MSC‐derived extracellular vesicles can also be mediated through regulation of endothelin signaling and endothelial matrix remodeling. huc‐MSC‐derived small extracellular vesicles (hUCMSC‐sEVs) reduced BSCB permeability and improved neurological recovery after SCI while suppressing prepro‐endothelin‐1 (prepro‐ET‐1) and ET‐1 expression. In vitro, hUCMSC‐sEVs restored endothelial junction proteins in oxygen‐glucose deprivation‐injured endothelial cells and reduced MMP‐2 and MMP‐9 expression through inhibition of ET‐1 signaling.^71^ Further mechanistic analysis identified exosomal miR‐149 as an important regulatory component. hUCMSC‐sEVs enriched in miR‐149 enhanced endothelial survival, restored tight and adherens junction proteins, reduced barrier permeability, and suppressed ET‐1 expression and PI3K/Akt activation. Overexpression of miR‐149 strengthened these effects, whereas miR‐149 inhibition abolished the protective response. In vivo, miR‐149‐enriched hUCMSC‐sEVs reduced BSCB leakage and tissue damage and improved locomotor recovery after SCI. The protective effect was associated with the miR‐149/ET‐1/PI3K/Akt axis.^72^ These studies collectively indicate that exosomal microRNAs can regulate multiple aspects of endothelial homeostasis, including junctional integrity, extracellular matrix remodeling, and intracellular survival signaling.

Exosome‐mediated vascular protection may also be enhanced through combination therapy. BMSC‐Exos combined with sodium tanshinone IIA sulfonate synergistically restored BSCB integrity by activating vascular endothelial growth factor signaling, promoting angiogenesis and endothelial cell migration, and reducing glial scar formation.^73^ These findings suggest that exosomes can serve as biological platforms that complement pharmacological agents, allowing simultaneous modulation of vascular regeneration and the inhibitory lesion environment. Such combination strategies may be particularly valuable because BSCB repair after SCI requires both stabilization of existing vessels and reconstruction of functional vascular networks.

In addition to repairing the BSCB, engineered exosomes may use the barrier itself as a therapeutic target. A multifunctional delivery system based on human UCMSC‐derived exosomes was modified with transactivator of transcription (TAT) and RVG peptides and loaded with a glutathione peroxidase 4 (GPX4) activator to improve BSCB penetration and spinal cord targeting. The engineered exosomes activated GPX4, reduced lipid peroxidation, regulated iron homeostasis, and suppressed ferroptosis and neuroinflammation. In SCI models, this targeted system protected neurons from ferroptotic death, promoted axonal regeneration, and improved the injured spinal cord microenvironment. These findings extend the role of exosome engineering beyond barrier repair itself, demonstrating that exosomes can be modified to facilitate therapeutic cargo delivery across or toward the injured neurovascular interface and subsequently target secondary injury mechanisms.^74^ It is reported that NSC‐derived exosomes containing ultrafine selenium nanoparticles (SeNExo) crossed the BSCB and blood‐brain barrier via the APOE‐LRP1 pathway, scavenged ROS, alleviated neuronal apoptosis, restored glial homeostasis, and rewired glial‐neuron networks.^75^ Although this approach represents a more extensively engineered exosome platform than conventional stem cell‐derived exosomes, it illustrates an important principle for SCI therapy: exosome‐based systems may function not only as barrier‐protective agents but also as vehicles capable of traversing or targeting the neurovascular barrier to deliver therapeutic cargo to the injured spinal cord.

Altogether, current evidence indicates that stem cell‐derived exosomes maintain or restore BSCB integrity through several converging mechanisms, including protection of pericytes from pyroptosis, preservation of endothelial junctions, inhibition of MMP‐mediated extracellular matrix degradation, regulation of ET‐1 and NF‐κB signaling, promotion of angiogenesis, and modulation of endothelial survival pathways. Exosomal miRNAs such as miR‐149 and miR‐501‐5p further demonstrate that specific non‐coding RNAs can directly regulate vascular barrier function. Meanwhile, ligand modification, intranasal administration, and biomaterial‐assisted delivery provide opportunities to improve vascular targeting, local retention, and therapeutic efficacy. Importantly, BSCB restoration may have effects that extend beyond vascular protection: limiting edema and peripheral immune‐cell infiltration can reduce secondary neuroinflammation, protect axons and oligodendrocytes, and create a more favorable environment for sensory pathway repair and neuropathic pain resolution.

## 6 SUMMARY OF KEY PRECLINICAL STUDIES

Table 1 provides a structured synthesis of representative studies included in this review, organized by therapeutic domain, exosome source, key molecular cargo or mechanism, experimental model, and primary outcome.

**Table 1:** Summary of representative stem cell‐derived exosome studies in SCI.

Domain	Exosome source	Key cargo/mechanism	Model	Primary outcome
Neuroinflammation	Dental pulp SC	ROS‐MAPK‐NFκB p65 inhibition	Rat SCI	↓ M1 macrophage polarization; ↓ secondary damage^24^
Neuroinflammation	HUCMSC	miR‐340‐5p/JAK‐STAT3	Rat SCI (in vivo/vitro)	↓ M1 microglia; ↓ neuroinflammation^25^
Neuroinflammation	BMSC	miR‐137 overexpression	Rat SCI	↓ Tissue damage; ↓ apoptosis; ↑ motor function^26^
Neuroinflammation	BMSC	circ_003564/NLRP3 pyroptosis	Rat SCI; PC12 cells	↓ Caspase‐1, GSDMD, IL‐1β, IL‐18; ↑ recovery^33^
Axonal regeneration	MSC	miR‐133b	Rat SCI	↑ Axonal regeneration; ↑ hindlimb recovery^34^
Axonal regeneration	BMSC (NGF‐overexpressing)	NGF; NSC differentiation	Mouse SCI	↑ Neuronal differentiation; ↑ axon growth^37^
Axonal regeneration	Engineered BMSC	miR‐17‐5p inhibitor/IL‐11/GP130/STAT3	Rat DRG; SCI	↑ Axonal growth; restored sensory conduction^9^
Ferroptosis	MSC	Nrf2/GCH1/BH4 axis	Rat SCI	↓ Microglial ferroptosis; ↑ motor function^28^
Ferroptosis	BMSC	miR‐219‐5p/UBE2Z/NRF2	Rat SCI	↓ Fe^2+^, MDA; ↑ GSH, SOD; ↑ motor function^32^
Ferroptosis	NSC	miR‐218a‐5p/Bmi1‐Mettl3‐Alox12	Rat SCI; PC12 cells	↓ Ferroptosis; ↑ motor function^30^
Neuropathic pain	HUCMSC	Gelfoam scaffold delivery	Rat SCI	↓ Neuropathic pain (1‐8 wks post‐op)^62^
Neuropathic pain	HUCMSC	Intrathecal delivery; TLR2/MyD88/NF‐κB ↓ Rsad2	Rat nerve ligation	↓ Mechanical allodynia; ↓ microglial activation^61^
BSCB integrity	CD146^+^CD271^+^ UCMSC	miR‐501‐5p/MLCK axis (RGD‐targeted)	Rat SCI	↑ Tight junction proteins; ↓ vascular leakage^38^
BSCB integrity	HUCMSC (sEV)	miR‐149/ET‐1/PI3K‐Akt	Rat SCI; endothelial OGD	↑ Barrier integrity; ↓ tissue damage^72^

Abbreviations: BMSC, bone marrow mesenchymal stem cell; BSCB, blood‐spinal cord barrier; DRG, dorsal root ganglion; GSH, glutathione; HUCMSC, human umbilical cord mesenchymal stem cell; MSC, mesenchymal stem cell; MDA, malondialdehyde; NSC, neural stem cell; OGD, oxygen‐glucose deprivation; SC, stem cell; SCI, spinal cord injury; sEV, small extracellular vesicle; SOD, superoxide dismutase; UCMSC, umbilical cord mesenchymal stem cell.

## 7 CURRENT CHALLENGES AND LIMITATIONS

Despite the promising preclinical evidence summarized above, several significant challenges must be addressed before stem cell‐derived exosomes can be translated to clinical practice.

First, the absence of standardized protocols for exosome isolation, purification, and large‐scale production represents a critical bottleneck. Currently used methods—including ultracentrifugation, size‐exclusion chromatography, and precipitation‐based approaches—yield products of variable purity, concentration, and biological activity, making cross‐study comparisons difficult and quality control for clinical‐grade (GMP) manufacturing challenging.

Second, functional heterogeneity among exosomes derived from different stem cell sources (e.g., BMSC, AD‐MSC, HUCMSC, NSC) and under different culture conditions (normoxic vs. hypoxic preconditioning; growth factor stimulation) complicates the selection of optimal cell sources for specific clinical indications. The molecular cargo—and therefore the therapeutic effect—of exosomes is highly sensitive to the physiological state of the parent cell.

Third, most studies investigating the therapeutic effects of stem cell‐derived exosomes in SCI have primarily focused on the restoration of motor function, with motor behavioral outcomes serving as the predominant measures of treatment efficacy. In contrast, studies specifically addressing sensory function restoration remain relatively limited. Besides, restoration of sensory function, particularly pain pathways, requires careful monitoring to avoid the risk of chronic pain arising from aberrant neural circuit formation. Exosome‐mediated axonal sprouting that reconnects sensory circuits inappropriately may inadvertently amplify allodynia or hyperalgesia. The current evidence for sensory recovery after SCI is largely derived from pain‐related behavioral assays, including mechanical allodynia and thermal hyperalgesia, whereas direct assessments of physiological sensory restoration remain limited. Improvements in these measures may indicate attenuation of neuropathic pain or pathological sensory hypersensitivity rather than complete restoration of normal sensory modalities. Objective and modality‐specific assessments of touch, proprioception, and temperature discrimination are therefore needed to establish whether stem cell‐derived exosomes can truly restore sensory function after SCI. Future studies should incorporate standardized and comprehensive sensory outcome measures alongside pain‐related behavioral assessments to distinguish genuine sensory recovery from the alleviation of neuropathic pain.

Fourth, the optimal delivery route remains unclear. Intravenous, intrathecal, intranasal, and local scaffold‐based delivery have all been used, but no systematic head‐to‐head comparisons across injury severities and time points have been published. Biodistribution, retention at the injury site, and off‐target effects differ substantially across routes.

Fifth, all published evidence to date derives from animal models (predominantly rodents). The translation from rodent SCI to human SCI is complicated by differences in spinal cord anatomy, injury biomechanics, immune responses, and the substantially longer distances over which axonal regeneration must occur in humans.

## 8 FUTURE DIRECTIONS

Future research should prioritize several interconnected areas to advance the clinical translation of stem cell exosome therapy for SCI.

Development of tissue‐engineered scaffolds for sustained and localized exosome delivery is a high‐priority direction. Hydrogels, electrospun fibers, and three‐dimensional composite scaffolds that release exosomes in a controlled fashion over days to weeks would overcome the limitations of single‐bolus injection and allow phase‐specific treatment (anti‐inflammatory in the acute phase; pro‐regenerative in the subacute and chronic phases).

Genetic engineering of exosomes to enrich specific neurotrophic factors, miRNAs, or proteins offers a rational approach to enhancing therapeutic potency and specificity. Surface modification with targeting ligands (e.g., RGD, RVG, ILP peptides) has already demonstrated improved BSCB penetration and lesion‐site accumulation and should be extended to optimize pharmacokinetics.

Stage‐specific precision delivery strategies—distinguishing between the acute inflammatory phase (Days 0–3), subacute phase (Days 4–14), and chronic phase (weeks to months)—should be developed. Different molecular targets predominate at each stage, and a single exosome formulation optimized for acute injury may be suboptimal or even counterproductive in the chronic setting.

Large animal models (pig, primate) with spinal cord anatomies more comparable to humans should be employed before clinical trials. Additionally, validated non‐invasive biomarkers (e.g., serum miRNA profiles, diffusion tensor imaging) of axonal integrity and BSCB permeability are needed to monitor treatment responses in future human studies.

Finally, the field would benefit from international consortia establishing consensus standards for exosome characterization (size distribution, surface markers, cargo profiling, potency assays) as prerequisites for regulatory approval, analogous to existing minimal criteria for MSC characterization.

## 9 CONCLUSIONS

SCI remains a major therapeutic challenge, as recovery of neurological function extends beyond motor improvement to include sensory deficits and neuropathic pain. Stem cell‐derived exosomes have emerged as promising cell‐free therapeutics with multifunctional effects on neuroprotection, immunomodulation, axonal regeneration, myelin repair, sensory restoration, pain modulation, and BSCB preservation. Increasing evidence suggests that stem cell‐derived exosomes may simultaneously promote structural repair and reshape the inflammatory, metabolic, and vascular microenvironment after SCI, providing a potential therapeutic strategy for more comprehensive neurological recovery. However, their clinical translation remains limited by heterogeneity in exosome production and characterization, insufficient standardization, uncertain biodistribution and delivery, and the predominance of preclinical evidence. In particular, sensory‐specific functional outcomes and long‐term effects on neuropathic pain require further investigation. Future studies integrating exosome engineering, targeted delivery, and biomaterial‐based platforms may improve therapeutic efficacy and safety. Collectively, stem cell‐derived exosomes represent a promising avenue for expanding SCI treatment beyond motor recovery toward integrated sensory restoration and pain management.

## AUTHOR CONTRIBUTIONS

Md Miftahul Mithu contributed to the conceptualization of the review, literature review and preparation of the original draft. Sahiduzzaman, Shirsendu Narayan Roy, John Jacob Mathew, Devunipalli Karthik Haraveer, Basundhari Thongbam, Kancharllapalli Prasanna Kumar, and Sourov Dey contributed to literature review, original drafting and editing of the manuscript. Md Arifuzzaman contributed to supervision, conceptualization, review and editing. All authors contributed to the manuscript, critically reviewed the content, and approved the final version for publication.

## CONFLICT OF INTEREST STATEMENT

The authors declare no conflict of interest.

## ETHICS STATEMENT

Not applicable as this is a review article.

## Data Availability

Data availability is not applicable to this review, as no new datasets were generated or analyzed during the current study. All data discussed in this review were obtained from previously published studies, which are appropriately cited in the manuscript.
